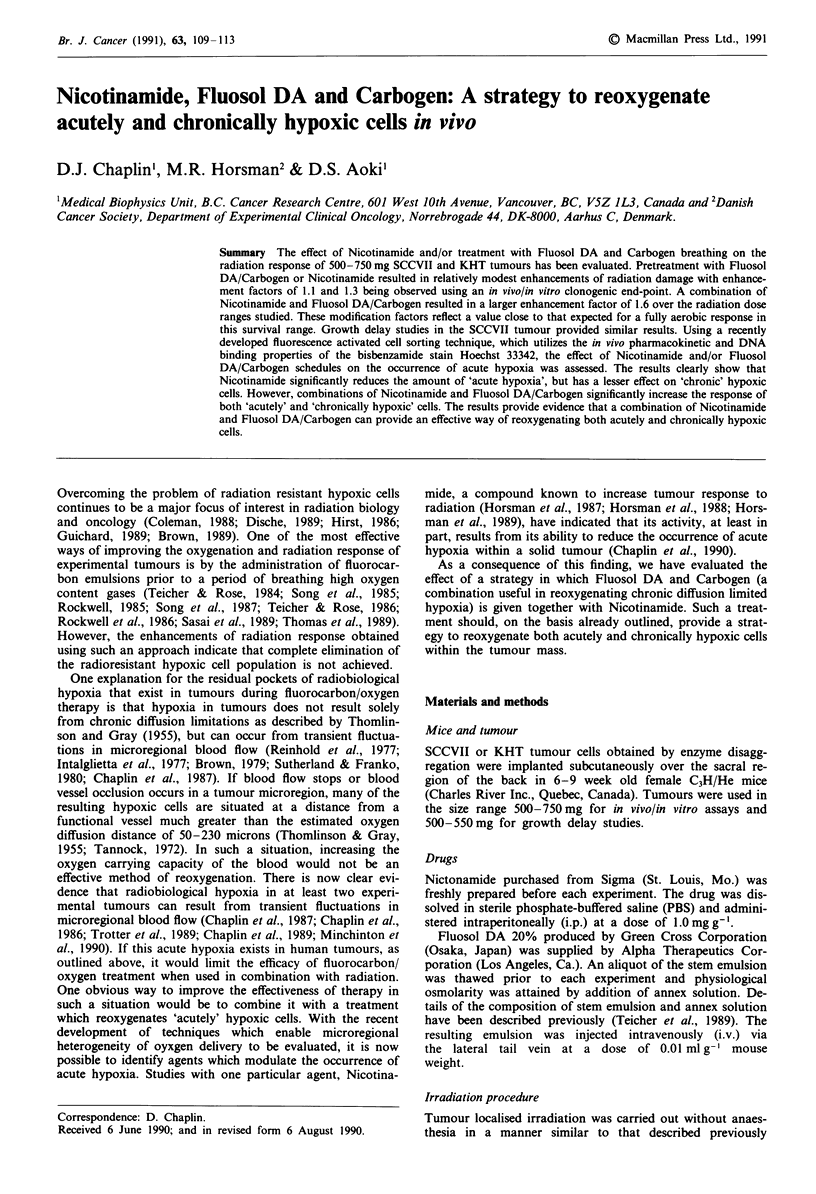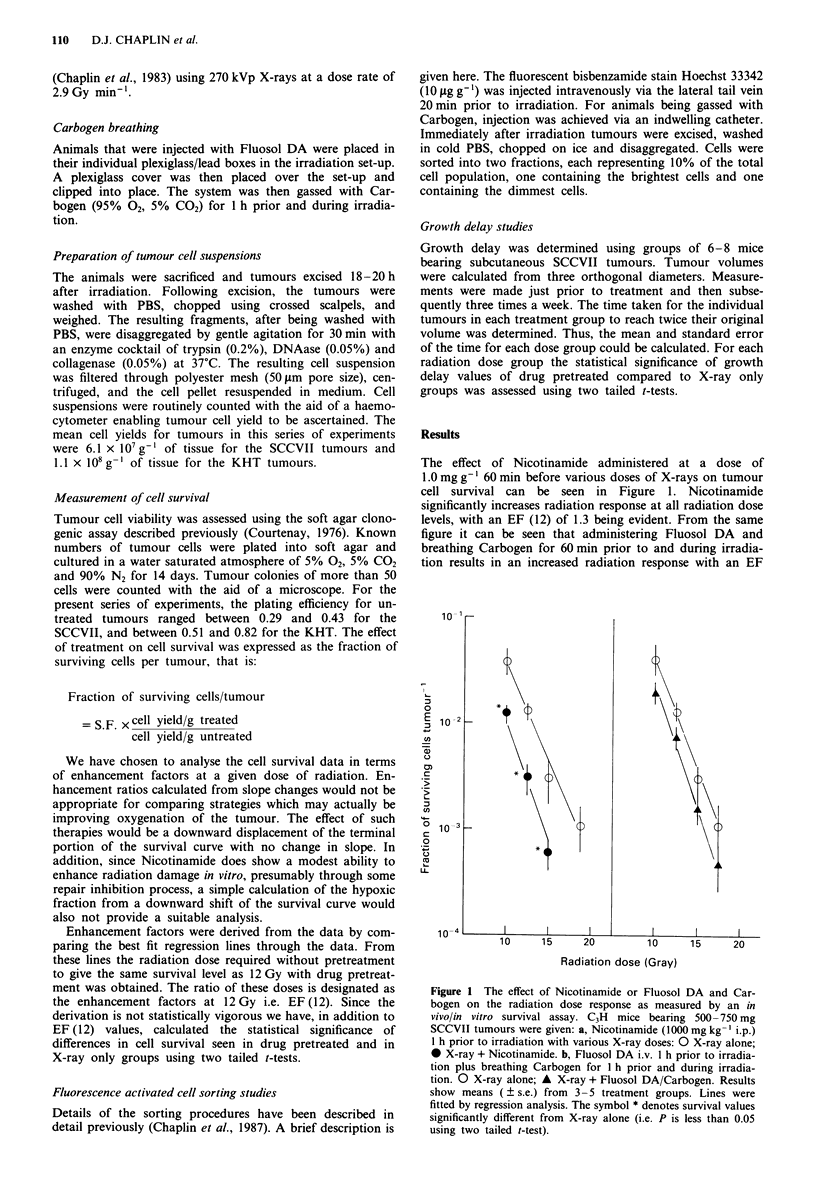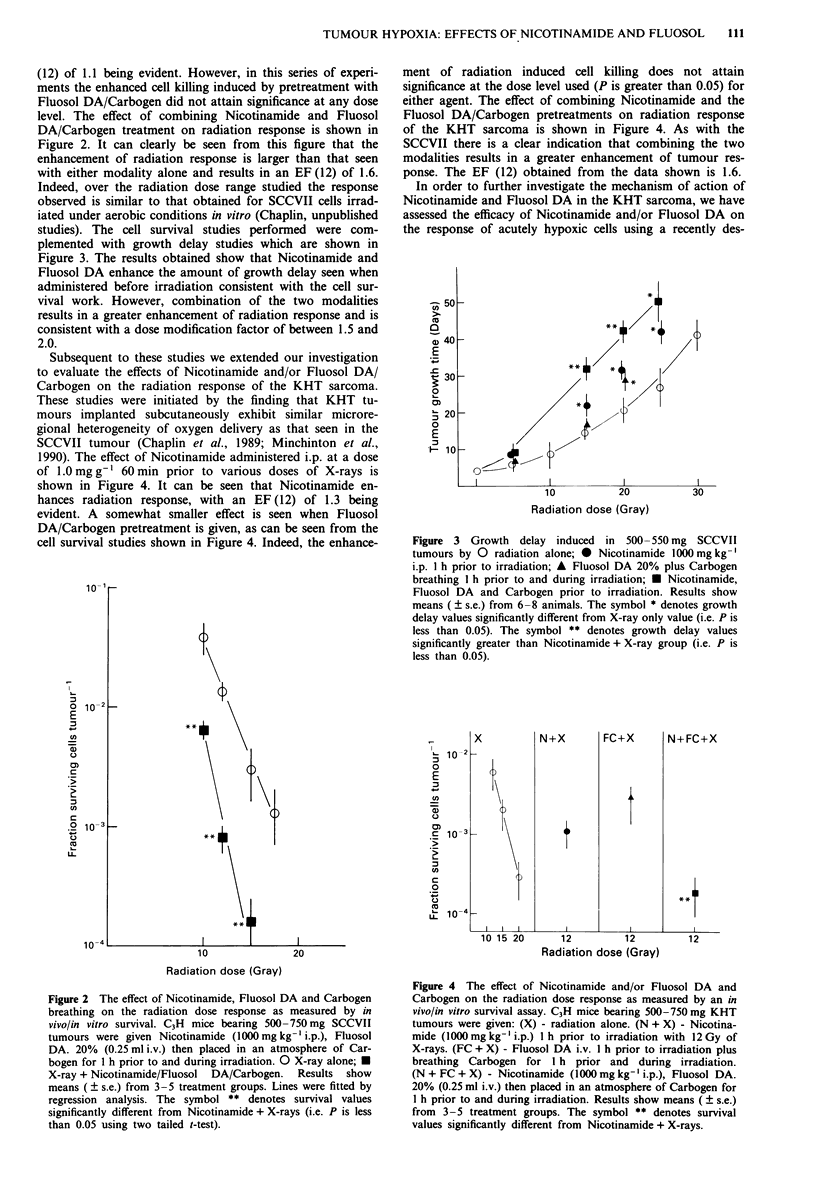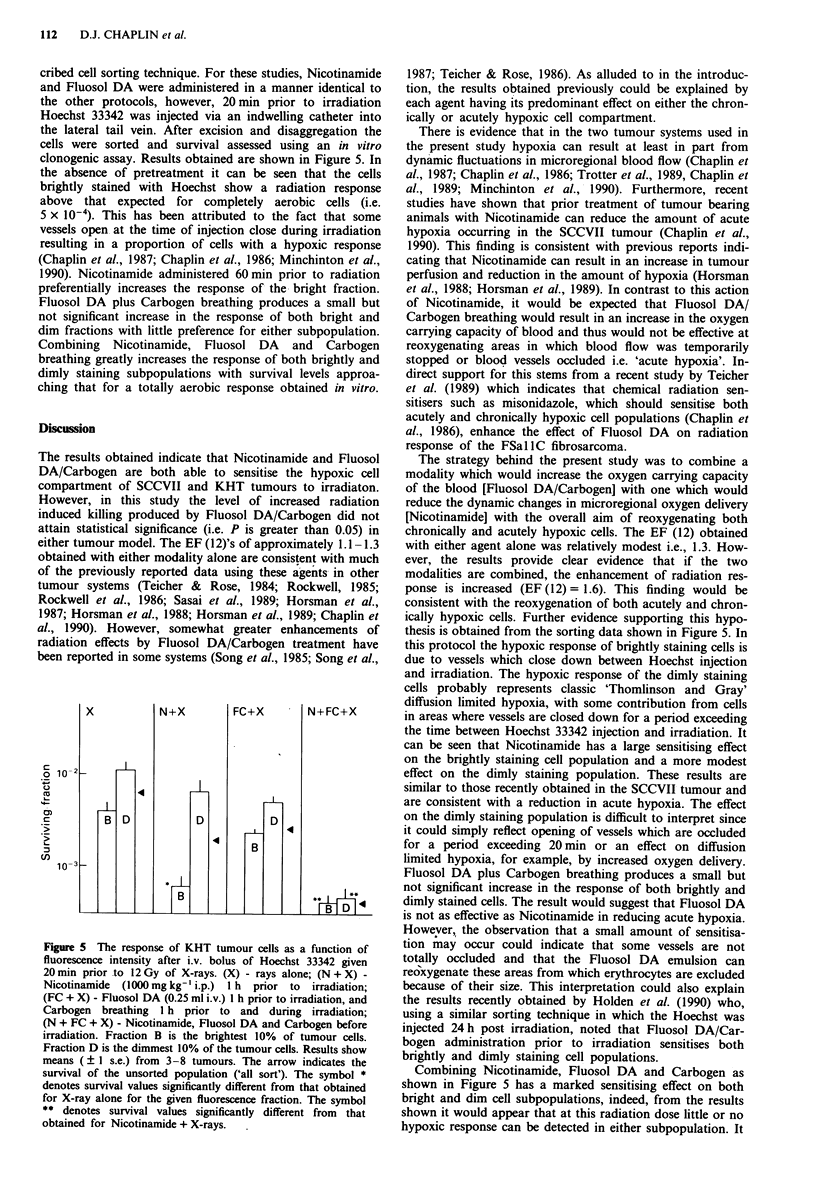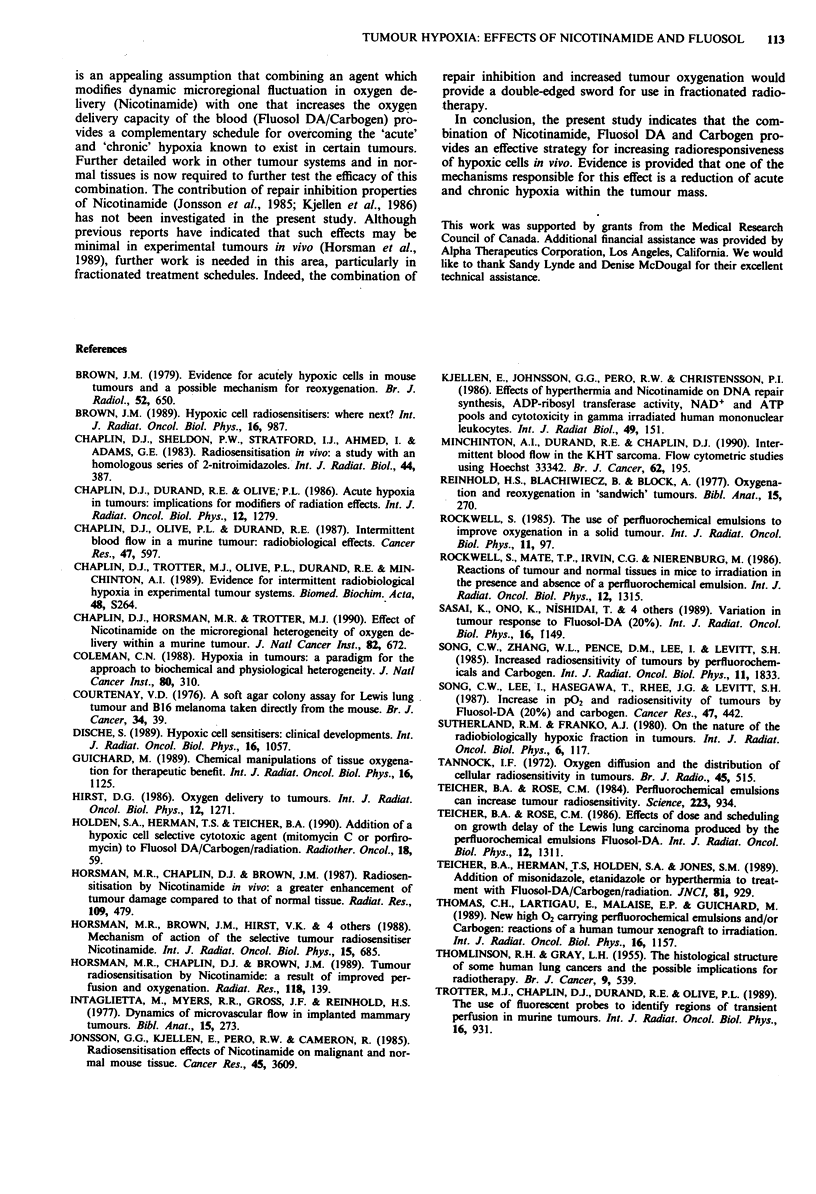# Nicotinamide, Fluosol DA and Carbogen: a strategy to reoxygenate acutely and chronically hypoxic cells in vivo.

**DOI:** 10.1038/bjc.1991.22

**Published:** 1991-01

**Authors:** D. J. Chaplin, M. R. Horsman, D. S. Aoki

**Affiliations:** Medical Biophysics Unit, B.C. Cancer Research Centre, Vancouver, Canada.

## Abstract

The effect of Nicotinamide and/or treatment with Fluosol DA and Carbogen breathing on the radiation response of 500-750 mg SCCVII and KHT tumours has been evaluated. Pretreatment with Fluosol DA/Carbogen or Nicotinamide resulted in relatively modest enhancements of radiation damage with enhancement factors of 1.1 and 1.3 being observed using an in vivo/in vitro clonogenic end-point. A combination of Nicotinamide and Fluosol DA/Carbogen resulted in a larger enhancement factor of 1.6 over the radiation dose ranges studied. These modification factors reflect a value close to that expected for a fully aerobic response in this survival range. Growth delay studies in the SCCVII tumour provided similar results. Using a recently developed fluorescence activated cell sorting technique, which utilizes the in vivo pharmacokinetic and DNA binding properties of the bisbenzamide stain Hoechst 33342, the effect of Nicotinamide and/or Fluosol DA/Carbogen schedules on the occurrence of acute hypoxia was assessed. The results clearly show that Nicotinamide significantly reduces the amount of 'acute hypoxia', but has a lesser effect on 'chronic' hypoxic cells. However, combinations of Nicotinamide and Fluosol DA/Carbogen significantly increase the response of both 'acutely' and 'chronically hypoxic' cells. The results provide evidence that a combination of Nicotinamide and Fluosol DA/Carbogen can provide an effective way of reoxygenating both acutely and chronically hypoxic cells.


Br. J. Cancer (1991), 63, 109-113 © Macmillan Press Ltd., 1991

### Nicotinamide, Fluosol DA and Carbogen: A strategy to reoxygenate acutely and chronically hypoxic cells *in vivo*

D.J. Chaplin[1], M.R. Horsman[2] & D.S. Aoki[1]

[1]*Medical Biophysics Unit, B.C. Cancer Research Centre, 601 West 10th Avenue, Vancouver, BC, V5Z 1L3, Canada and* [2]*Danish Cancer Society, Department of Experimental Clinical Oncology, Norrebrogade 44, DK-8000, Aarhus C, Denmark.*

**Summary** The effect of Nicotinamide and/or treatment with Fluosol DA and Carbogen breathing on the radiation response of 500-750 mg SCCVII and KHT tumours has been evaluated. Pretreatment with Fluosol DA/Carbogen or Nicotinamide resulted in relatively modest enhancements of radiation damage with enhancement factors of 1.1 and 1.3 being observed using an *in vivo/in vitro* clonogenic end-point. A combination of Nicotinamide and Fluosol DA/Carbogen resulted in a larger enhancement factor of 1.6 over the radiation dose ranges studied. These modification factors reflect a value close to that expected for a fully aerobic response in this survival range. Growth delay studies in the SCCVII tumour provided similar results. Using a recently developed fluorescence activated cell sorting technique, which utilizes the *in vivo* pharmacokinetic and DNA binding properties of the bisbenzamide stain Hoechst 33342, the effect of Nicotinamide and/or Fluosol DA/Carbogen schedules on the occurrence of acute hypoxia was assessed. The results clearly show that Nicotinamide significantly reduces the amount of 'acute hypoxia', but has a lesser effect on 'chronic' hypoxic cells. However, combinations of Nicotinamide and Fluosol DA/Carbogen significantly increase the response of both 'acutely' and 'chronically hypoxic' cells. The results provide evidence that a combination of Nicotinamide and Fluosol DA/Carbogen can provide an effective way of reoxygenating both acutely and chronically hypoxic cells.

Overcoming the problem of radiation resistant hypoxic cells continues to be a major focus of interest in radiation biology and oncology (Coleman, 1988; Dische, 1989; Hirst, 1986; Guichard, 1989; Brown, 1989). One of the most effective ways of improving the oxygenation and radiation response of experimental tumours is by the administration of fluorocarbon emulsions prior to a period of breathing high oxygen content gases (Teicher & Rose, 1984; Song *et al.*, 1985; Rockwell, 1985; Song *et al.*, 1987; Teicher & Rose, 1986; Rockwell *et al.*, 1986; Sasai *et al.*, 1989; Thomas *et al.*, 1989). However, the enhancements of radiation response obtained using such an approach indicate that complete elimination of the radioresistant hypoxic cell population is not achieved.

One explanation for the residual pockets of radiobiological hypoxia that exist in tumours during fluorocarbon/oxygen therapy is that hypoxia in tumours does not result solely from chronic diffusion limitations as described by Thomlinson and Gray (1955), but can occur from transient fluctuations in microregional blood flow (Reinhold *et al.*, 1977; Intalglietta *et al.*, 1977; Brown, 1979; Sutherland & Franko, 1980; Chaplin *et al.*, 1987). If blood flow stops or blood vessel occlusion occurs in a tumour microregion, many of the resulting hypoxic cells are situated at a distance from a functional vessel much greater than the estimated oxygen diffusion distance of 50-230 microns (Thomlinson & Gray, 1955; Tannock, 1972). In such a situation, increasing the oxygen carrying capacity of the blood would not be an effective method of reoxygenation. There is now clear evidence that radiobiological hypoxia in at least two experimental tumours can result from transient fluctuations in microregional blood flow (Chaplin *et al.*, 1987; Chaplin *et al.*, 1986; Trotter *et al.*, 1989; Chaplin *et al.*, 1989; Minchinton *et al.*, 1990). If this acute hypoxia exists in human tumours, as outlined above, it would limit the efficacy of fluorocarbon/oxygen treatment when used in combination with radiation. One obvious way to improve the effectiveness of therapy in such a situation would be to combine it with a treatment which reoxygenates 'acutely' hypoxic cells. With the recent development of techniques which enable microregional heterogeneity of oyxgen delivery to be evaluated, it is now possible to identify agents which modulate the occurrence of acute hypoxia. Studies with one particular agent, Nicotinamide, a compound known to increase tumour response to radiation (Horsman *et al.*, 1987; Horsman *et al.*, 1988; Horsman *et al.*, 1989), have indicated that its activity, at least in part, results from its ability to reduce the occurrence of acute hypoxia within a solid tumour (Chaplin *et al.*, 1990).

As a consequence of this finding, we have evaluated the effect of a strategy in which Fluosol DA and Carbogen (a combination useful in reoxygenating chronic diffusion limited hypoxia) is given together with Nicotinamide. Such a treatment should, on the basis already outlined, provide a strategy to reoxygenate both acutely and chronically hypoxic cells within the tumour mass.

### Materials and methods

### Mice and tumour

SCCVII or KHT tumour cells obtained by enzyme disaggregation were implanted subcutaneously over the sacral region of the back in 6-9 week old female $C_3H$/He mice (Charles River Inc., Quebec, Canada). Tumours were used in the size range 500-750 mg for *in vivo/in vitro* assays and 500-550 mg for growth delay studies.

### Drugs

Nictonamide purchased from Sigma (St. Louis, Mo.) was freshly prepared before each experiment. The drug was dissolved in sterile phosphate-buffered saline (PBS) and administered intraperitoneally (i.p.) at a dose of $1.0 \text{ mg g}^{-1}$.

Fluosol DA 20% produced by Green Cross Corporation (Osaka, Japan) was supplied by Alpha Therapeutics Corporation (Los Angeles, Ca.). An aliquot of the stem emulsion was thawed prior to each experiment and physiological osmolarity was attained by addition of annex solution. Details of the composition of stem emulsion and annex solution have been described previously (Teicher *et al.*, 1989). The resulting emulsion was injected intravenously (i.v.) via the lateral tail vein at a dose of $0.01 \text{ ml g}^{-1}$ mouse weight.

### Irradiation procedure

Tumour localised irradiation was carried out without anaesthesia in a manner similar to that described previously

Correspondence: D. Chaplin.
Received 6 June 1990; and in revised form 6 August 1990.

(Chaplin *et al.*, 1983) using 270 kVp X-rays at a dose rate of 2.9 Gy $min^{-1}$.

*Carbogen breathing*

Animals that were injected with Fluosol DA were placed in their individual plexiglass/lead boxes in the irradiation set-up. A plexiglass cover was then placed over the set-up and clipped into place. The system was then gassed with Carbogen (95% $O_2$, 5% $CO_2$) for 1 h prior and during irradiation.

*Preparation of tumour cell suspensions*

The animals were sacrificed and tumours excised 18–20 h after irradiation. Following excision, the tumours were washed with PBS, chopped using crossed scalpels, and weighed. The resulting fragments, after being washed with PBS, were disaggregated by gentle agitation for 30 min with an enzyme cocktail of trypsin (0.2%), DNAase (0.05%) and collagenase (0.05%) at 37°C. The resulting cell suspension was filtered through polyester mesh (50 μm pore size), centrifuged, and the cell pellet resuspended in medium. Cell suspensions were routinely counted with the aid of a haemocytometer enabling tumour cell yield to be ascertained. The mean cell yields for tumours in this series of experiments were $6.1 \times 10^7$ $g^{-1}$ of tissue for the SCCVII tumours and $1.1 \times 10^8$ $g^{-1}$ of tissue for the KHT tumours.

*Measurement of cell survival*

Tumour cell viability was assessed using the soft agar clonogenic assay described previously (Courtenay, 1976). Known numbers of tumour cells were plated into soft agar and cultured in a water saturated atmosphere of 5% $O_2$, 5% $CO_2$ and 90% $N_2$ for 14 days. Tumour colonies of more than 50 cells were counted with the aid of a microscope. For the present series of experiments, the plating efficiency for untreated tumours ranged between 0.29 and 0.43 for the SCCVII, and between 0.51 and 0.82 for the KHT. The effect of treatment on cell survival was expressed as the fraction of surviving cells per tumour, that is:

$$\text{Fraction of surviving cells/tumour} = \text{S.F.} \times \frac{\text{cell yield/g treated}}{\text{cell yield/g untreated}}$$

We have chosen to analyse the cell survival data in terms of enhancement factors at a given dose of radiation. Enhancement ratios calculated from slope changes would not be appropriate for comparing strategies which may actually be improving oxygenation of the tumour. The effect of such therapies would be a downward displacement of the terminal portion of the survival curve with no change in slope. In addition, since Nicotinamide does show a modest ability to enhance radiation damage *in vitro*, presumably through some repair inhibition process, a simple calculation of the hypoxic fraction from a downward shift of the survival curve would also not provide a suitable analysis.

Enhancement factors were derived from the data by comparing the best fit regression lines through the data. From these lines the radiation dose required without pretreatment to give the same survival level as 12 Gy with drug pretreatment was obtained. The ratio of these doses is designated as the enhancement factors at 12 Gy i.e. EF (12). Since the derivation is not statistically vigorous we have, in addition to EF (12) values, calculated the statistical significance of differences in cell survival seen in drug pretreated and in X-ray only groups using two tailed *t*-tests.

*Fluorescence activated cell sorting studies*

Details of the sorting procedures have been described in detail previously (Chaplin *et al.*, 1987). A brief description is given here. The fluorescent bisbenzamide stain Hoechst 33342 (10 μg $g^{-1}$) was injected intravenously via the lateral tail vein 20 min prior to irradiation. For animals being gassed with Carbogen, injection was achieved via an indwelling catheter. Immediately after irradiation tumours were excised, washed in cold PBS, chopped on ice and disaggregated. Cells were sorted into two fractions, each representing 10% of the total cell population, one containing the brightest cells and one containing the dimmest cells.

*Growth delay studies*

Growth delay was determined using groups of 6–8 mice bearing subcutaneous SCCVII tumours. Tumour volumes were calculated from three orthogonal diameters. Measurements were made just prior to treatment and then subsequently three times a week. The time taken for the individual tumours in each treatment group to reach twice their original volume was determined. Thus, the mean and standard error of the time for each dose group could be calculated. For each radiation dose group the statistical significance of growth delay values of drug pretreated compared to X-ray only groups was assessed using two tailed *t*-tests.

### Results

The effect of Nicotinamide administered at a dose of 1.0 mg $g^{-1}$ 60 min before various doses of X-rays on tumour cell survival can be seen in Figure 1. Nicotinamide significantly increases radiation response at all radiation dose levels, with an EF (12) of 1.3 being evident. From the same figure it can be seen that administering Fluosol DA and breathing Carbogen for 60 min prior to and during irradiation results in an increased radiation response with an EF

**Figure 1** The effect of Nicotinamide or Fluosol DA and Carbogen on the radiation dose response as measured by an *in vivo/in vitro* survival assay. $C_3H$ mice bearing 500–750 mg SCCVII tumours were given: **a**, Nicotinamide (1000 mg $kg^{-1}$ i.p.) 1 h prior to irradiation with various X-ray doses: ○ X-ray alone; ● X-ray + Nicotinamide. **b**, Fluosol DA i.v. 1 h prior to irradiation plus breathing Carbogen for 1 h prior and during irradiation. ○ X-ray alone; ▲ X-ray + Fluosol DA/Carbogen. Results show means (± s.e.) from 3–5 treatment groups. Lines were fitted by regression analysis. The symbol * denotes survival values significantly different from X-ray alone (i.e. *P* is less than 0.05 using two tailed *t*-test).

(12) of 1.1 being evident. However, in this series of experiments the enhanced cell killing induced by pretreatment with Fluosol DA/Carbogen did not attain significance at any dose level. The effect of combining Nicotinamide and Fluosol DA/Carbogen treatment on radiation response is shown in Figure 2. It can clearly be seen from this figure that the enhancement of radiation response is larger than that seen with either modality alone and results in an EF (12) of 1.6. Indeed, over the radiation dose range studied the response observed is similar to that obtained for SCCVII cells irradiated under aerobic conditions *in vitro* (Chaplin, unpublished studies). The cell survival studies performed were complemented with growth delay studies which are shown in Figure 3. The results obtained show that Nicotinamide and Fluosol DA enhance the amount of growth delay seen when administered before irradiation consistent with the cell survival work. However, combination of the two modalities results in a greater enhancement of radiation response and is consistent with a dose modification factor of between 1.5 and 2.0.

Subsequent to these studies we extended our investigation to evaluate the effects of Nicotinamide and/or Fluosol DA/Carbogen on the radiation response of the KHT sarcoma. These studies were initiated by the finding that KHT tumours implanted subcutaneously exhibit similar microregional heterogeneity of oxygen delivery as that seen in the SCCVII tumour (Chaplin *et al.*, 1989; Minchinton *et al.*, 1990). The effect of Nicotinamide administered i.p. at a dose of 1.0 mg $g^{-1}$ 60 min prior to various doses of X-rays is shown in Figure 4. It can be seen that Nicotinamide enhances radiation response, with an EF (12) of 1.3 being evident. A somewhat smaller effect is seen when Fluosol DA/Carbogen pretreatment is given, as can be seen from the cell survival studies shown in Figure 4. Indeed, the enhancement of radiation induced cell killing does not attain significance at the dose level used (*P* is greater than 0.05) for either agent. The effect of combining Nicotinamide and the Fluosol DA/Carbogen pretreatments on radiation response of the KHT sarcoma is shown in Figure 4. As with the SCCVII there is a clear indication that combining the two modalities results in a greater enhancement of tumour response. The EF (12) obtained from the data shown is 1.6.

In order to further investigate the mechanism of action of Nicotinamide and Fluosol DA in the KHT sarcoma, we have assessed the efficacy of Nicotinamide and/or Fluosol DA on the response of acutely hypoxic cells using a recently des-

**Figure 2** The effect of Nicotinamide, Fluosol DA and Carbogen breathing on the radiation dose response as measured by *in vivo/in vitro* survival. $C_3H$ mice bearing 500–750 mg SCCVII tumours were given Nicotinamide (1000 mg $kg^{-1}$ i.p.), Fluosol DA. 20% (0.25 ml i.v.) then placed in an atmosphere of Carbogen for 1 h prior to and during irradiation. O X-ray alone; ■ X-ray + Nicotinamide/Fluosol DA/Carbogen. Results show means ( ± s.e.) from 3–5 treatment groups. Lines were fitted by regression analysis. The symbol ** denotes survival values significantly different from Nicotinamide + X-rays (i.e. *P* is less than 0.05 using two tailed *t*-test).

**Figure 3** Growth delay induced in 500–550 mg SCCVII tumours by O radiation alone; ● Nicotinamide 1000 mg $kg^{-1}$ i.p. 1 h prior to irradiation; ▲ Fluosol DA 20% plus Carbogen breathing 1 h prior to and during irradiation; ■ Nicotinamide, Fluosol DA and Carbogen prior to irradiation. Results show means ( ± s.e.) from 6–8 animals. The symbol * denotes growth delay values significantly different from X-ray only value (i.e. *P* is less than 0.05). The symbol ** denotes growth delay values significantly greater than Nicotinamide + X-ray group (i.e. *P* is less than 0.05).

**Figure 4** The effect of Nicotinamide and/or Fluosol DA and Carbogen on the radiation dose response as measured by an *in vivo/in vitro* survival assay. $C_3H$ mice bearing 500–750 mg KHT tumours were given: (X) - radiation alone. (N + X) - Nicotinamide (1000 mg $kg^{-1}$ i.p.) 1 h prior to irradiation with 12 Gy of X-rays. (FC + X) - Fluosol DA i.v. 1 h prior to irradiation plus breathing Carbogen for 1 h prior and during irradiation. (N + FC + X) - Nicotinamide (1000 mg $kg^{-1}$ i.p.), Fluosol DA. 20% (0.25 ml i.v.) then placed in an atmosphere of Carbogen for 1 h prior to and during irradiation. Results show means ( ± s.e.) from 3–5 treatment groups. The symbol ** denotes survival values significantly different from Nicotinamide + X-rays.

cribed cell sorting technique. For these studies, Nicotinamide and Fluosol DA were administered in a manner identical to the other protocols, however, 20 min prior to irradiation Hoechst 33342 was injected via an indwelling catheter into the lateral tail vein. After excision and disaggregation the cells were sorted and survival assessed using an *in vitro* clonogenic assay. Results obtained are shown in Figure 5. In the absence of pretreatment it can be seen that the cells brightly stained with Hoechst show a radiation response above that expected for completely aerobic cells (i.e. $5 \times 10^{-4}$). This has been attributed to the fact that some vessels open at the time of injection close during irradiation resulting in a proportion of cells with a hypoxic response (Chaplin *et al.*, 1987; Chaplin *et al.*, 1986; Minchinton *et al.*, 1990). Nicotinamide administered 60 min prior to radiation preferentially increases the response of the bright fraction. Fluosol DA plus Carbogen breathing produces a small but not significant increase in the response of both bright and dim fractions with little preference for either subpopulation. Combining Nicotinamide, Fluosol DA and Carbogen breathing greatly increases the response of both brightly and dimly staining subpopulations with survival levels approaching that for a totally aerobic response obtained *in vitro*.

### Discussion

The results obtained indicate that Nicotinamide and Fluosol DA/Carbogen are both able to sensitise the hypoxic cell compartment of SCCVII and KHT tumours to irradiaton. However, in this study the level of increased radiation induced killing produced by Fluosol DA/Carbogen did not attain statistical significance (i.e. $P$ is greater than 0.05) in either tumour model. The EF (12)'s of approximately 1.1–1.3 obtained with either modality alone are consistent with much of the previously reported data using these agents in other tumour systems (Teicher & Rose, 1984; Rockwell, 1985; Rockwell *et al.*, 1986; Sasai *et al.*, 1989; Horsman *et al.*, 1987; Horsman *et al.*, 1988; Horsman *et al.*, 1989; Chaplin *et al.*, 1990). However, somewhat greater enhancements of radiation effects by Fluosol DA/Carbogen treatment have been reported in some systems (Song *et al.*, 1985; Song *et al.*, 1987; Teicher & Rose, 1986). As alluded to in the introduction, the results obtained previously could be explained by each agent having its predominant effect on either the chronically or acutely hypoxic cell compartment.

**Figure 5** The response of KHT tumour cells as a function of fluorescence intensity after i.v. bolus of Hoechst 33342 given 20 min prior to 12 Gy of X-rays. (X) - rays alone; (N + X) - Nicotinamide (1000 mg kg$^{-1}$ i.p.) 1 h prior to irradiation; (FC + X) - Fluosol DA (0.25 ml i.v.) 1 h prior to irradiation, and Carbogen breathing 1 h prior to and during irradiation; (N + FC + X) - Nicotinamide, Fluosol DA and Carbogen before irradiation. Fraction B is the brightest 10% of tumour cells. Fraction D is the dimmest 10% of the tumour cells. Results show means (± 1 s.e.) from 3–8 tumours. The arrow indicates the survival of the unsorted population ('all sort'). The symbol * denotes survival values significantly different from that obtained for X-ray alone for the given fluorescence fraction. The symbol ** denotes survival values significantly different from that obtained for Nicotinamide + X-rays.

There is evidence that in the two tumour systems used in the present study hypoxia can result at least in part from dynamic fluctuations in microregional blood flow (Chaplin *et al.*, 1987; Chaplin *et al.*, 1986; Trotter *et al.*, 1989, Chaplin *et al.*, 1989; Minchinton *et al.*, 1990). Furthermore, recent studies have shown that prior treatment of tumour bearing animals with Nicotinamide can reduce the amount of acute hypoxia occurring in the SCCVII tumour (Chaplin *et al.*, 1990). This finding is consistent with previous reports indicating that Nicotinamide can result in an increase in tumour perfusion and reduction in the amount of hypoxia (Horsman *et al.*, 1988; Horsman *et al.*, 1989). In contrast to this action of Nicotinamide, it would be expected that Fluosol DA/Carbogen breathing would result in an increase in the oxygen carrying capacity of blood and thus would not be effective at reoxygenating areas in which blood flow was temporarily stopped or blood vessels occluded i.e. 'acute hypoxia'. Indirect support for this stems from a recent study by Teicher *et al.* (1989) which indicates that chemical radiation sensitisers such as misonidazole, which should sensitise both acutely and chronically hypoxic cell populations (Chaplin *et al.*, 1986), enhance the effect of Fluosol DA on radiation response of the FSa11C fibrosarcoma.

The strategy behind the present study was to combine a modality which would increase the oxygen carrying capacity of the blood [Fluosol DA/Carbogen] with one which would reduce the dynamic changes in microregional oxygen delivery [Nicotinamide] with the overall aim of reoxygenating both chronically and acutely hypoxic cells. The EF (12) obtained with either agent alone was relatively modest i.e., 1.3. However, the results provide clear evidence that if the two modalities are combined, the enhancement of radiation response is increased (EF (12) = 1.6). This finding would be consistent with the reoxygenation of both acutely and chronically hypoxic cells. Further evidence supporting this hypothesis is obtained from the sorting data shown in Figure 5. In this protocol the hypoxic response of brightly staining cells is due to vessels which close down between Hoechst injection and irradiation. The hypoxic response of the dimly staining cells probably represents classic 'Thomlinson and Gray' diffusion limited hypoxia, with some contribution from cells in areas where vessels are closed down for a period exceeding the time between Hoechst 33342 injection and irradiation. It can be seen that Nicotinamide has a large sensitising effect on the brightly staining cell population and a more modest effect on the dimly staining population. These results are similar to those recently obtained in the SCCVII tumour and are consistent with a reduction in acute hypoxia. The effect on the dimly staining population is difficult to interpret since it could simply reflect opening of vessels which are occluded for a period exceeding 20 min or an effect on diffusion limited hypoxia, for example, by increased oxygen delivery. Fluosol DA plus Carbogen breathing produces a small but not significant increase in the response of both brightly and dimly stained cells. The result would suggest that Fluosol DA is not as effective as Nicotinamide in reducing acute hypoxia. However, the observation that a small amount of sensitisation may occur could indicate that some vessels are not totally occluded and that the Fluosol DA emulsion can reoxygenate these areas from which erythrocytes are excluded because of their size. This interpretation could also explain the results recently obtained by Holden *et al.* (1990) who, using a similar sorting technique in which the Hoechst was injected 24 h post irradiation, noted that Fluosol DA/Carbogen administration prior to irradiation sensitises both brightly and dimly staining cell populations.

Combining Nicotinamide, Fluosol DA and Carbogen as shown in Figure 5 has a marked sensitising effect on both bright and dim cell subpopulations, indeed, from the results shown it would appear that at this radiation dose little or no hypoxic response can be detected in either subpopulation. It is an appealing assumption that combining an agent which modifies dynamic microregional fluctuation in oxygen delivery (Nicotinamide) with one that increases the oxygen delivery capacity of the blood (Fluosol DA/Carbogen) provides a complementary schedule for overcoming the 'acute' and 'chronic' hypoxia known to exist in certain tumours. Further detailed work in other tumour systems and in normal tissues is now required to further test the efficacy of this combination. The contribution of repair inhibition properties of Nicotinamide (Jonsson *et al*., 1985; Kjellen *et al*., 1986) has not been investigated in the present study. Although previous reports have indicated that such effects may be minimal in experimental tumours *in vivo* (Horsman *et al*., 1989), further work is needed in this area, particularly in fractionated treatment schedules. Indeed, the combination of repair inhibition and increased tumour oxygenation would provide a double-edged sword for use in fractionated radiotherapy.

In conclusion, the present study indicates that the combination of Nicotinamide, Fluosol DA and Carbogen provides an effective strategy for increasing radioresponsiveness of hypoxic cells *in vivo*. Evidence is provided that one of the mechanisms responsible for this effect is a reduction of acute and chronic hypoxia within the tumour mass.

This work was supported by grants from the Medical Research Council of Canada. Additional financial assistance was provided by Alpha Therapeutics Corporation, Los Angeles, California. We would like to thank Sandy Lynde and Denise McDougal for their excellent technical assistance.

### References

BROWN, J.M. (1979). Evidence for acutely hypoxic cells in mouse tumours and a possible mechanism for reoxygenation. *Br. J. Radiol*., **52**, 650.

BROWN, J.M. (1989). Hypoxic cell radiosensitisers: where next? *Int. J. Radiat. Oncol. Biol. Phys*., **16**, 987.

CHAPLIN, D.J., SHELDON, P.W., STRATFORD, I.J., AHMED, I. & ADAMS, G.E. (1983). Radiosensitisation *in vivo*: a study with an homologous series of 2-nitroimidazoles. *Int. J. Radiat. Biol*., **44**, 387.

CHAPLIN, D.J., DURAND, R.E. & OLIVE, P.L. (1986). Acute hypoxia in tumours: implications for modifiers of radiation effects. *Int. J. Radiat. Oncol. Biol. Phys*., **12**, 1279.

CHAPLIN, D.J., OLIVE, P.L. & DURAND, R.E. (1987). Intermittent blood flow in a murine tumour: radiobiological effects. *Cancer Res*., **47**, 597.

CHAPLIN, D.J., TROTTER, M.J., OLIVE, P.L., DURAND, R.E. & MINCHINTON, A.I. (1989). Evidence for intermittent radiobiological hypoxia in experimental tumour systems. *Biomed. Biochim. Acta*, **48**, S264.

CHAPLIN, D.J., HORSMAN, M.R. & TROTTER, M.J. (1990). Effect of Nicotinamide on the microregional heterogeneity of oxygen delivery within a murine tumour. *J. Natl Cancer Inst*., **82**, 672.

COLEMAN, C.N. (1988). Hypoxia in tumours: a paradigm for the approach to biochemical and physiological heterogeneity. *J. Natl Cancer Inst*., **80**, 310.

COURTENAY, V.D. (1976). A soft agar colony assay for Lewis lung tumour and B16 melanoma taken directly from the mouse. *Br. J. Cancer*, **34**, 39.

DISCHE, S. (1989). Hypoxic cell sensitisers: clinical developments. *Int. J. Radiat. Oncol. Biol. Phys*., **16**, 1057.

GUICHARD, M. (1989). Chemical manipulations of tissue oxygenation for therapeutic benefit. *Int. J. Radiat. Oncol. Biol. Phys*., **16**, 1125.

HIRST, D.G. (1986). Oxygen delivery to tumours. *Int. J. Radiat. Oncol. Biol. Phys*., **12**, 1271.

HOLDEN, S.A., HERMAN, T.S. & TEICHER, B.A. (1990). Addition of a hypoxic cell selective cytotoxic agent (mitomycin C or porfiromycin) to Fluosol DA/Carbogen/radiation. *Radiother. Oncol*., **18**, 59.

HORSMAN, M.R., CHAPLIN, D.J. & BROWN, J.M. (1987). Radiosensitisation by Nicotinamide *in vivo*: a greater enhancement of tumour damage compared to that of normal tissue. *Radiat. Res*., **109**, 479.

HORSMAN, M.R., BROWN, J.M., HIRST, V.K. & 4 others (1988). Mechanism of action of the selective tumour radiosensitiser Nicotinamide. *Int. J. Radiat. Oncol. Biol. Phys*., **15**, 685.

HORSMAN, M.R., CHAPLIN, D.J. & BROWN, J.M. (1989). Tumour radiosensitisation by Nicotinamide: a result of improved perfusion and oxygenation. *Radiat. Res*., **118**, 139.

INTAGLIETTA, M., MYERS, R.R., GROSS, J.F. & REINHOLD, H.S. (1977). Dynamics of microvascular flow in implanted mammary tumours. *Bibl. Anat*., **15**, 273.

JONSSON, G.G., KJELLEN, E., PERO, R.W. & CAMERON, R. (1985). Radiosensitisation effects of Nicotinamide on malignant and normal mouse tissue. *Cancer Res*., **45**, 3609.

KJELLEN, E., JOHNSSON, G.G., PERO, R.W. & CHRISTENSSON, P.I. (1986). Effects of hyperthermia and Nicotinamide on DNA repair synthesis, ADP-ribosyl transferase activity, $NAD^+$ and ATP pools and cytotoxicity in gamma irradiated human mononuclear leukocytes. *Int. J. Radiat Biol*., **49**, 151.

MINCHINTON, A.I., DURAND, R.E. & CHAPLIN, D.J. (1990). Intermittent blood flow in the KHT sarcoma. Flow cytometric studies using Hoechst 33342. *Br. J. Cancer*, **62**, 195.

REINHOLD, H.S., BLACHIWIECZ, B. & BLOCK, A. (1977). Oxygenation and reoxygenation in 'sandwich' tumours. *Bibl. Anat*., **15**, 270.

ROCKWELL, S. (1985). The use of perfluorochemical emulsions to improve oxygenation in a solid tumour. *Int. J. Radiat. Oncol. Biol. Phys*., **11**, 97.

ROCKWELL, S., MATE, T.P., IRVIN, C.G. & NIERENBURG, M. (1986). Reactions of tumour and normal tissues in mice to irradiation in the presence and absence of a perfluorochemical emulsion. *Int. J. Radiat. Oncol. Biol. Phys*., **12**, 1315.

SASAI, K., ONO, K., NISHIDAI, T. & 4 others (1989). Variation in tumour response to Fluosol-DA (20%). *Int. J. Radiat. Oncol. Biol. Phys*., **16**, 1149.

SONG, C.W., ZHANG, W.L., PENCE, D.M., LEE, I. & LEVITT, S.H. (1985). Increased radiosensitivity of tumours by perfluorochemicals and Carbogen. *Int. J. Radiat. Oncol. Biol. Phys*., **11**, 1833.

SONG, C.W., LEE, I., HASEGAWA, T., RHEE, J.G. & LEVITT, S.H. (1987). Increase in $pO_2$ and radiosensitivity of tumours by Fluosol-DA (20%) and carbogen. *Cancer Res*., **47**, 442.

SUTHERLAND, R.M. & FRANKO, A.J. (1980). On the nature of the radiobiologically hypoxic fraction in tumours. *Int. J. Radiat. Oncol. Biol. Phys*., **6**, 117.

TANNOCK, I.F. (1972). Oxygen diffusion and the distribution of cellular radiosensitivity in tumours. *Br. J. Radio*., **45**, 515.

TEICHER, B.A. & ROSE, C.M. (1984). Perfluorochemical emulsions can increase tumour radiosensitivity. *Science*, **223**, 934.

TEICHER, B.A. & ROSE, C.M. (1986). Effects of dose and scheduling on growth delay of the Lewis lung carcinoma produced by the perfluorochemical emulsions Fluosol-DA. *Int. J. Radiat. Oncol. Biol. Phys*., **12**, 1311.

TEICHER, B.A., HERMAN, T.S, HOLDEN, S.A. & JONES, S.M. (1989). Addition of misonidazole, etanidazole or hyperthermia to treatment with Fluosol-DA/Carbogen/radiation. *JNCI*, **81**, 929.

THOMAS, C.H., LARTIGAU, E., MALAISE, E.P. & GUICHARD, M. (1989). New high $O_2$ carrying perfluorochemical emulsions and/or Carbogen: reactions of a human tumour xenograft to irradiation. *Int. J. Radiat. Oncol. Biol. Phys*., **16**, 1157.

THOMLINSON, R.H. & GRAY, L.H. (1955). The histological structure of some human lung cancers and the possible implications for radiotherapy. *Br. J. Cancer*, **9**, 539.

TROTTER, M.J., CHAPLIN, D.J., DURAND, R.E. & OLIVE, P.L. (1989). The use of fluorescent probes to identify regions of transient perfusion in murine tumours. *Int. J. Radiat. Oncol. Biol. Phys*., **16**, 931.